# The Epidemiology of Anal Human Papillomavirus (HPV) in HIV-Positive and HIV-Negative Women and Men: A Ten-Year Retrospective Observational Study in Rome (Italy)

**DOI:** 10.3390/pathogens13020163

**Published:** 2024-02-11

**Authors:** Matteo Fracella, Giuseppe Oliveto, Piergiorgio Roberto, Lilia Cinti, Massimo Gentile, Eleonora Coratti, Gabriella D’Ettorre, Eugenio Nelson Cavallari, Francesco Romano, Letizia Santinelli, Luca Maddaloni, Federica Frasca, Carolina Scagnolari, Guido Antonelli, Alessandra Pierangeli

**Affiliations:** 1 Virology Laboratory, Department of Molecular Medicine, Sapienza University, 00185 Rome, Italy; matteo.fracella@uniroma1.it (M.F.); giuseppe.oliveto@uniroma1.it (G.O.); massimo.gentile@uniroma1.it (M.G.); coratti.1847223@studenti.uniroma1.it (E.C.); federica.frasca@uniroma1.it (F.F.); carolina.scagnolari@uniroma1.it (C.S.); guido.antonelli@uniroma1.it (G.A.); 2 Microbiology and Virology Unit, Sapienza University Hospital Policlinico Umberto I, 00186 Rome, Italy; piergiorgio.roberto@uniroma1.it (P.R.); lilia.cinti@uniroma1.it (L.C.); 3 Department of Public Health and Infectious Diseases, Sapienza University, 00185 Rome, Italy; gabriella.dettorre@uniroma1.it (G.D.); eugenionelson.cavallari@uniroma1.it (E.N.C.); francesco.romano@uniroma1.it (F.R.); letizia.santinelli@uniroma1.it (L.S.); federica.frasca@uniroma1.it (F.F.)

**Keywords:** human papillomaviruses, anal HPV infection, oncogenic HPV, genotyping, HPV vaccine

## Abstract

Human papillomaviruses (HPVs) commonly infect the anogenital mucosa; most infections are transient, but a fraction of those caused by high-risk (HR) types persist and may lead to anogenital cancer. The epidemiology of HPV genotypes in anal infections in groups at different risk for anal cancer has not been well described in Italy. This retrospective study reports the results of HPV DNA testing and complete genotyping performed on anal swabs from 691 female and male patients attending proctology clinics in Rome during 2012–2021; one-third had repeated testing. Cumulative HPV positivity in 1212 anal swabs was approximately 60%, was not age related, and showed an increasing trend over the study period. HPV rates differed significantly by sex and HIV status: HIV-negative women had the lowest (43.6%) and HIV-positive men the highest (83.5%) HPV prevalence. HIV-positive men had more oncogenic HPV genotypes detected, more multiple infections, and the highest frequency of persistent infections. Two-thirds of all infections were vaccine-preventable. This study found that anal HPV infection rates are still elevated and even increasing in groups at low and high risk of developing anal cancer. Prevention programs need to be improved to reduce rates of anal infection in young women and men.

## 1. Introduction

Human papillomaviruses (HPVs) are part of the large *Papillomaviridae* family that have evolved to replicate in the stratified epithelia of various body sites [1]. HPV genotypes are classified taxonomically based on genomic divergence of the gene encoding the major capsid protein L1 [2]. To date, more than 200 HPVs have been identified that show mucosal or cutaneous tropism and approximately 40 HPV genotypes of the alpha genus commonly infect the anogenital and oral mucosa. Among the high-risk (HR) HPVs, 12 genotypes (HPV16, 18, 31, 33, 35, 39, 45, 51, 52, 56, 58, and 59) have been classified by IARC [3] as Group 1, labeled “Carcinogenic to Humans”, HPV68 has been classified as “Probably Carcinogenic”, in Group 2A, while another set of 12 genotypes (HPV26, 53, 66, 67, 70, 73, 82, 30, 34, 69, 85, and 97) have been classified as “possibly carcinogenic to Humans” in Group 2B. Similar to its oncogenic role in the cervix, HPV plays a causal role in the development of over 90% of squamous cell carcinomas of the anal canal, with HPV16 being the most common cause [4,5]. HPV-associated anal cancer is still increasing in high-income countries [6,7,8], and in the United States, anal cancer may become more frequent than cervical cancer in unvaccinated older women [9]. In addition, HPV causes anal warts (condyloma acuminate) that cause discomfort and pain, and their persistence is associated with a higher risk of developing anal cancer [10]. HPV vaccination has been long proven to be effective in preventing anal infections and HPV-related lesions [11] and is expected to reduce the risk of anal cancer in vaccinated individuals. However, a recent meta-analysis showed that vaccine efficacy may be restricted to HIV-negative individuals vaccinated at ≤26 years of age [12].

In Italy, HPV vaccination was introduced for adolescent girls in 2008 and for adolescent and high-risk males in 2015–2018, depending on regional health services; vaccination coverage in girls reached a maximum of about 70% in 2013 (birth cohort 2001) and then started to decline, while coverage in males peaked at almost 50% in 2019 (birth cohort 2001) [13]. Although the overall prevalence of high-risk anal HPV infections has been widely reported, especially among HIV-infected men and men who have sex with men (MSM) [14,15,16,17], the epidemiology of HPV genotypes in groups at lower risk of anal cancer, such as HIV-negative women, has not been well described in Italy. Such knowledge would be of great value in understanding the effectiveness of vaccination programs over time. 

Therefore, the objectives of this study were to describe the prevalence of anal HPV infection over time in patients attending proctology visits for suspected anal disease, to describe the specific infecting genotypes in women and in HIV-negative and HIV-positive men, and to compare the outcome of anal HPV infection in multiple tested individuals between groups.

## 2. Materials and Methods

### 2.1. Study Design

This observational study involved anal brushing samples performed for diagnostic purpose for HPV DNA testing on patients consecutively attending the proctological clinic and the Department of Infectious Diseases of the Policlinico Umberto I, Sapienza University Hospital, Rome, from January 2012 to December 2021. The Sapienza University proctological clinic is a specialized clinic that performs screening visits, sampling for diagnostic services, and treatments for anal lesions; reasons to visit the proctological clinic are symptoms such as bleeding, itchiness, or hemorrhoids, and HPV testing on the anal swab was performed in the presence of a clinical suspicion of HPV-related lesions. HIV-positive patients are seen at the Department of Infectious Diseases for periodic visits, and they are subjected to anal brushing if they present with symptoms indicative of anal lesions. All HIV-positive patients tested for HPV were receiving ART and were virologically suppressed. 

The HPV DNA tests were performed at the Virology laboratory of the Department of Molecular Medicine. Most patients had only one anal sample, whereas others returned over the time for whom two or more anal HPV DNA test were available. 

The patient’s consent was not asked as the HPV DNA test was a diagnostic procedure; this retrospective study was approved by the institutional ethic committee of Sapienza University (Rif. 6963, Prot. 0055/2023).

### 2.2. HPV Detection and Genotyping

Anal specimens were centrifuged at a low speed; the cell pellets then underwent DNA extraction and PCR amplification using the consensus primer MY09/11 targeting a 450 bp fragment from the HPV L1 region, as previously described [18]. PCR products were subjected to Sanger sequencing; resulting sequences in Fasta format, derived from automatic base calling only, were compared with those in GenBank using the NCBI Blast tool [19]. The following criteria were used to assign the genotype: minimum sequence coverage of 75%, percentage of identity of at least 88% with the specific HPV genotype, and e-value < 2 × 10^−51^. Mixed chromatograms in which no single genotype could be identified were considered indicative of the presence of co-infecting HPVs (multiple infection).

In this study, HPV genotypes were classified according to the IARC classification [3] into Group 1 (HPV16, 18, 31, 33, 35, 39, 45, 51, 52, 56, 58, and 59), Group 2A/2B (HPV68 and HPV26, 53, 66, 67, 70, 73, 82, 30, 34, 69, 85, and 97), and low-risk (LR) HPVs, including HPV6 and 11, which are classified in IARC Group 3 (unclassifiable as to carcinogenicity in humans), the other mucosal genotypes that are epidemiologically considered LR HPVs [2], and the detected cutaneous genotypes of the Beta genus (HPV38, 107, 110, 113, 120, and 145).

### 2.3. Data Analysis 

Pearson χ^2^ test and Mantel–Haenszel odds ratio (OR) values were calculated using SPSS software (IBM, Corporation, Armonk, NY, USA) version 24.0. Statistical significance was set at *p* < 0.05. 

## 3. Results

### 3.1. Prevalence of HPV Genotypes in Anal Samples

This observational study included anal swab specimens (N = 1212) obtained for diagnostic purposes for HPV DNA testing in female and male patients attending the proctology clinic and the Department of Infectious Diseases of the Policlinico Umberto I Hospital, Rome, from January 2012 to December 2021. 

Anal specimens were collected from a total of 691 enrolled patients, of which N = 438 (63.4%) had HPV testing on a single anal specimen, while the others (N = 253) returned for proctological visits over time and had more than one HPV DNA test. Among patients who were retested for HPV DNA, N = 106 had two tests, N = 70 had three, N = 50 had four, N = 10 had five, and N = 17 had a total of six HPV DNA tests.

Of the 691 patients tested, 500/691 (72.4%) were male and 191/691 (27.6%) were female; the median age was similar by sex (42.9 years in men and 43.1 years in women). During the study period, 462/691 (66.9%) were positive for HPV DNA at least once. Of note, the HPV positivity rate was significantly (*p* < 0.001) higher in males than in females (377/500: 75.4% and 85/191: 44.5%, respectively), did not differ by patient age, and was similar across three age groups: ≤35 y, 35–50 y, >50 y (Table 1). HIV status was known for 617 subjects: N = 411/617 were HIV-negative (66.6%) and N = 206/617 were HIV-positive (33.4%). HPV positivity was significantly (*p* < 0.001) higher in HIV-positive than in HIV-negative patients (169/206: 82.0% and 237/411: 57.7%, respectively). The anal HPV prevalence by sex, age, and HIV status is summarized in Table 1.

**Figure 1:**
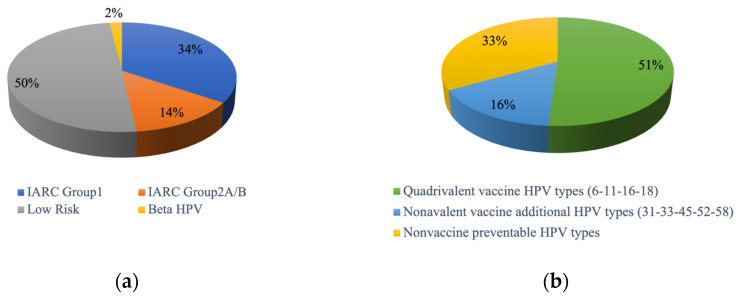
Distribution of identified genotypes in HPV-positive anal samples (N = 660) according to (**a**) IARC classification and (**b**) vaccine composition.

The cumulative HPV positivity rate for the total number of samples tested was 58.8% (N = 713/1212). Among the HPV-positive tests, N = 53/713 (7.4%) could not be typed probably due to mixed infections; of the 660 samples with an identified genotype, 227/660 (34.4%) were carcinogenic HPVs of the IARC Group 1, 90/660 (13.6%) were of Groups 2A or 2B, 329/660 (49.8%) were LR-HPVs, and 14/660 (2.1%) were cutaneous genotypes of the Beta genus (Figure 1a).

Nine genotypes were the most commonly detected in the total anal samples: HPV6 (N = 148/660, 22.4%), HPV16 (N = 97/660, 14.7%), HPV11 (N = 71/660, 10.8%), HPV58 (N = 40/660, 6.6%), HPV53 (N = 31/660, 4.7%), HPV61 (N = 25/660, 3.8%), HPV31 (N = 24/660, 3.6%), HPV33 (N = 21/660, 3.2%), and HPV18 (N = 20/660, 2.7%); the frequency of all other genotypes was below 2.5%. 

The identified genotypes were classified as either preventable or not preventable by the HPV vaccines. Approximately half of the cases (51%) were positive for the four types included in the quadrivalent vaccine (HPV6, 11, 16, 18), and an additional 16% were positive for the five additional genotypes preventable with the nonavalent vaccine (HPV31, 33, 45, 52, and 58) (Figure 1b).

### 3.2. HPV Distribution According to Sex and HIV Status

Because it is well established that sex and HIV status have an impact on the acquisition and persistence of anal HPV infection, we sought to analyze the HPV epidemiology in our study group by comparing patients based on these characteristics. The HIV status was known for all female (N = 191) and 426/500 (85.2%) male patients; only 12/191 women (6.3%) were HIV-positive, and of these, 7/12 (58.3%) were HPV-positive. Because of the small number of HIV-positive women, we excluded them from subsequent analyses and compared HPV data among the HIV-negative women (hereafter referred to as women, N = 179), the HIV-negative men (N = 232), and the HIV-positive men (N = 194), also excluding the 74 men with unknown HIV status.

The median age of patients in the three groups did not differ significantly (Table 2). The HPV-positive rate was significantly higher in HIV-positive men (83.5%) compared to the other two groups (*p* < 0.001, Table 2). Among HIV-negative patients, men were more likely to be HPV-positive than women (68.3% vs. 43.6%: *p* < 0.001). Similarly, the rate of any infection with carcinogenic HPVs (i.e., those belonging to IARC Group 1) was higher in HIV-positive men (30.9%) compared to HIV-negative men (23.3%) and women (16.2%) (*p* = 0.0036, Table 2).

A substantial proportion of enrolled patients (significantly higher in HIV-positive men, *p* = 0.0067, Table 2) had more than one HPV DNA test (two to six in all groups, with a mean interval of 14-16 months). The outcome of HPV infection was different in the repeat-tested patients of the three groups (*p* = 0.026, Table 2). In particular, HPV DNA clearance (defined as at least two HPV DNA negative tests after one HPV positive test) was less frequent, whereas persistent infection (defined as at least two HPV DNA tests performed more than one year apart that were positive for the same HPV genotype) was more frequent in the HIV-positive men compared with the other groups (Table 2); however, this persistence was caused by both carcinogenic and LR HPV genotypes.

**Figure 2:**
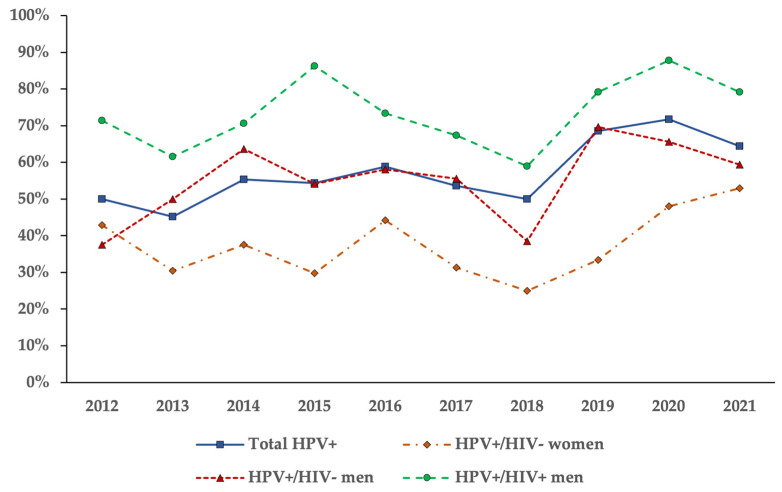
Distribution of HPV-positive results in anal specimens over ten consecutive years (2012–2021). The annual percentage of HPV-positive tests over the total number of tests is shown for the entire study group and for patients with known HIV status stratified in HIV-negative women, HIV-negative men, and HIV-positive men.

The rates of HPV infection over the study period and the distribution of HPV genotypes detected in the anal swabs were then compared among the HIV-negative women, HIV-negative men, and HIV-positive men. Figure 2 shows the annual rates of HPV positivity for the total anal swab specimens (N = 1212) and for patients with known HIV status stratified into the three groups. Considering the percentage of HPV-positive tests out of the total tests performed in a year, the HPV DNA positivity percentages fluctuated over the years, but cumulatively remained stable or even increased over time (Figure 2).

A total of 23 different HPV genotypes were found in 110 specimens from women, 27 genotypes in 222 specimens from HIV-negative men, and as many as 39 different genotypes in 320 specimens from HIV-positive men (Table 2). The latter group also had more non-typable genotypes (9%) than the other groups, suggesting a higher rate of multiple infections. The percentage of persistent infections differed between groups (*p* = 0.026); in particular, HIV-positive men had a fivefold higher odds ratio of developing HPV persistence compared with HIV-negative men (Table 2).

Among the nine genotypes most frequently detected in the total anal specimens described above, HPV11 detection differed significantly among the three groups (*p* = 0.004), being less frequent in women and more prevalent in the HIV-negative men (Table 2). There were no differences among the three groups in positivity for carcinogenic, probably/possibly carcinogenic, and LR HPVs (Table 2). The number of specimens with HPV16 and HPV18 detected (infections preventable with the bivalent HPV vaccine) did not differ between groups. In contrast, the distribution of genotypes preventable with the quadrivalent and nonavalent vaccines differed significantly between groups; in particular, infections preventable with these two vaccines were more common in the HIV-negative men (Table 2).

## 4. Discussion

For this observational study, we retrospectively collected and analyzed the results of HPV DNA testing performed on anal specimens from female and male patients attending proctology visits over a ten-year period. Among 691 patients enrolled, the cumulative prevalence of anal HPV infection was high: two-thirds were positive at least once during the study period. The individuals tested were symptomatic patients attending either a proctology or infectious disease clinic, so the high prevalence found is not representative of HPV infection rates in asymptomatic patients from the general population.

The HPV prevalence did not show an age-dependent trend but was significantly higher among men (75.4%) compared to women (44.5%). Potential biases influencing this sex difference in our cohort are lower willingness of women to be tested, lack of information on sexual orientation as MSM are more likely to have anal HPV infection [14,15,16,17] and the low representation of HIV-positive individuals among women. Indeed, excluding the 12 HIV-positive women, HPV was significantly more prevalent in HIV-positive men (83.5%) compared with HIV-negative men (68.3%) and HIV-negative women (43.6%).

In women, the HPV prevalence registered for the decade 2012–2021 (44.5%) is higher than that found in our previous study (34.7%) testing women attending the same proctology clinic and using the same HPV DNA method, from April 2005 to June 2011 [18]. To the best of our knowledge, no other Italian study has reported the anal HPV prevalence in HIV-negative women, but this rate is consistent with data from a large meta-analysis that reported an overall HPV prevalence in HIV-negative women ranging from 42% in normal cytology to 90% in anal cancer cells [20]. Furthermore, another collaborative meta-analysis showed that cervical HR-HPV positivity was associated with anal HR-HPV prevalence [21]; specifically, in HIV-negative women, the anal HR-HPV prevalence was 43% in cervical HR-HPV-positive women versus 9% in cervical HR-HPV-negative women. Cervical HPV positivity and cytology results were not known in our group of women to directly compare our results. However, the increasing trend in HPV anal infections among women compared to our previous work and observed during this study period is consistent with global studies that have observed an increase in sexual risk factors for HPV transmission [22,23], which are likely to counteract the effectiveness of vaccination in reducing HPV anal infections [11]. In addition, female vaccination coverage in Italy has been declining since 2014, and the COVID-19 pandemic further negatively affected HPV vaccination coverage in Italy in 2020 [24], both in girls and boys.

HIV-negative men had a high rate of HPV anal infection (68.3%), intermediate compared with women and HIV-positive men. In a study conducted between August 2009 and June 2017 at a center for sexually transmitted infections in Rome [17], 72.8% of 452 HIV-negative MSM were found to be HPV-positive; this rate is comparable to that found in the present study, considering the presence of an unknown but probably relevant proportion of MSM among our HIV-negative men. In the same Rome study [17], 95% of 321 HIV-positive men had anal HPV infection, which is a higher percentage compared to the 83.5% positivity found in the present study; different risk factors (e.g. sexual behavior, number of partners) could account for this difference. Our group previously detected 88.9% and 94.4% of HPV positivity in HIV-negative MSM and HIV-positive MSM, respectively [25]. A large meta-analysis [20] reported an overall HPV prevalence in HIV-negative men ranging from 57% in samples with normal cytology to 89% in cancer. Unfortunately, due to the retrospective nature of this study, it was not possible to know the patients’ risk factors for HPV acquisition, such as number of sex partners, their HPV status, and history of anal intercourse, nor were cytology results available. It is likely that if it had been possible to stratify men based on these risk factors, we would have observed significant differences in anal HPV prevalence. Indeed, HIV-negative men who have sex with women (MSW) have lower rates of anal HPV infection [26,27,28], compared with women and HIV-negative MSM.

HIV-positive men had the highest rate of HPV anal infection (83%) and of oncogenic genotypes (31%) among the groups, and a fivefold higher odds ratio of developing HPV persistence, consistent with previous literature indicating that this group is at high risk of developing HPV-associated anal lesions and cancer [4,5,27,28]. To lower this risk, in the Sapienza University Infectious Diseases Clinics, anal HPV testing has been performed during periodic visits in the presence of symptoms indicative of anal lesions since 2004 [25]; interestingly, recent guidelines recommended HR-HPV DNA testing as an acceptable screening tool for anal cancer starting at age 35 for HIV-positive MSM and at age 45 for other high-risk groups [29].

To characterize the epidemiology of anal HPV and to assess the potential impact of vaccination, the presence of specific HPV genotypes was determined using widely used PCR primers MY09-MY11 targeting an L1 genomic tract and genotypes identified by sequencing analysis. This method has the limitation that PCR amplification and sequencing techniques accurately detect the more abundant (and clinically relevant) type, but do not reveal co-infecting genotypes. In fact, the untyped HPV genotypes (about 7.5% of the total study specimens, 9% in the HIV-positive men) are likely to be mixed infections that cannot be resolved by sequencing, but these rates are far lower rates than those reported in the literature. In a recent meta-analysis, rates of mixed infection among HIV-positive men ranged from 61% among MSM to 40% among MSW, while among HIV-negative men they ranged from 43% among MSM to 13% among MSWs [28]. On the other hand, the broad-spectrum L1 PCR allows the identification of almost all mucosal HPVs and many alpha and beta genus HPVs with cutaneous tropism. In fact, genotype characterization by sequencing allowed us to report on the full spectrum of HPV types infecting the anal canal, distinguishing genotypes not targeted by commercial HPV DNA tests, in particular LR-HPVs and genotypes whose oncogenic risk is not well defined. In addition, complete genotyping allowed us to define HPV reinfection (positivity for different HPV genotypes in sequential samples) versus HPV persistence (positivity for the same HPV genotype in sequential samples taken more than one year apart). However, it is important to note that reinfection with the same genotype is possible, and because viral variants were not assessed, some reinfection could be misinterpreted as persistence.

Regarding the distribution of HPV genotypes in anal infections, the two most prevalent genotypes overall and in the three groups were HPV6 and HPV16. Certainly, HPV16 is the major cause of the global and local anal cancer burden [4,5,30] and is relatively more frequent in high-grade lesions than in cytology-negative and LSIL anal samples [20,27,28,30]. In men, the specific prevalence of HPV16 varies by HIV-status and sexual orientation, from 2–3% in HIV-negative MSW to around 30% in HIV-positive MSM [20,27,28,30]. In HIV-negative women, the more significant risk factor for anal HPV16 detection is concurrent cervical HPV16 infection; in a large collaborative meta-analysis, this rate varied from 41% in cervical HPV16-positive women to 2% in cervical HPV16-negative women [21]. The HPV6 prevalence is reported in a relatively low number of studies as it is not included in most commercial HPV DNA tests targeting HR genotypes. HPV6 was also the most frequently identified genotype in anal infections in our previous studies in women [18] and in HIV-negative and -positive men [25]. In a cross-sectional study of anal HPV prevalence conducted in Milan, Italy, among HIV-positive men [31], HPV6 was the second most common genotype after HPV16, but intriguingly, its frequency was almost as high as HPV16 in samples with positive anal cytology [31]. In our study, the third prevalent genotype, HPV11, had different rates between the groups, being detected more frequently in the HIV-negative men. Interestingly, IARC group 3 HPVs 6 and 11 are found in cancer cells in single infections and may play a role in anal carcinogenesis [10,20,32,33]. The fourth genotype preventable with the quadrivalent vaccine, HPV18, was detected in 2–3% of cases; together these four genotypes accounted for about half of the anal HPV infections in this study. Three other genotypes included in the nonavalent vaccine were detected in 3–6% of cases, HPV31, HPV33, and HPV58, while HPV45 was detected only in male patients and HPV52 was detected only in two HIV-positive men. The potential efficacy of the quadrivalent and nonavalent vaccines differed among the three groups and was significantly higher in the HIV-negative men. The vaccination status of the study groups is not known; we assume that the majority were not vaccinated as vaccination was introduced in Italy in 2008 and 2018 for adolescent females and males, respectively [30]. Overall, the nonavalent vaccine could have prevented two-thirds of the anal infections in our study group, not considering possible cross-protection against nonvaccine types. Therefore, a campaign should be conducted in Italy to increase vaccination coverage and to reinforce the concept that HPV vaccines could prevent HPV-related lesions other than cervical cancer. In fact, the possibility that HPV vaccination after treatment may also reduce the risk of anal disease recurrence has been proposed and positively evaluated [34,35].

Genotypes not covered by the HPV vaccines were detected in around 4% of anal specimens, namely HPV53 and HPV61. HPV53, classified in the IARC group 2B, is frequently found in cervical and anal infections in Italy [18] and worldwide [36,37] but its attributable (etiological) fraction in cancers caused by HPVs is near to zero [38]. Differently, HPV61 is detected only by broad-spectrum PCR and not by commercial methods; among our study groups, it was detected in 5% of the cases among women and HIV-positive men but only once in HIV-negative men. HPV61, which remains an unclassified risk by IARC, is rare in cancer but relatively common in LSIL [39], as also found in Italian women [40,41].

## 5. Conclusions

In conclusion, this study adds to the limited data on the prevalence of anal HPV infection in HIV-negative women and shows that anal HPV infection may be as high as 40%, even in a group with few known risk factors. In addition, we confirmed the high burden of anal HPV infection in men, especially in HIV-positive patients, in whom more oncogenic HPV genotypes, more multiple infections, and the highest frequency of persistent infections were detected. Therefore, there is a need to increase awareness of the risk of anal HPV infection and the benefits of vaccination in Italy. As a result of this broad characterization of the genotypes infecting the anal mucosa, we can conclude that the nonavalent vaccine has the potential to prevent anal cancer and at least two-thirds of anal infections, thus avoiding repeat testing, patient visits, and treatment for anal lesions.

## Figures and Tables

**Table 1:** Overall anal HPV prevalence by sex, age, and HIV status.

Tested Patients (N = 691)	Total	HPV DNA		*p*-Value
		Negative	Positive ^a^	
Male, N (%) Female, N (%)	500/691 (72.4) 191/691 (27.6)	123/500 (24.6) 106/191 (55.5)	377/500 (75.4) 85/191 (44.5)	<0.001
Age (years), median (range) ^b^	42.9 (15.5–86.1)	42.6 (17.7–86.1)	43 (15.5–80.7)	ns
Age Group ^b^				
≤35 y, N (%)	189/639 (29.6)	66/189 (34.9)	123/189 (65.1)	ns
35–50 y, N (%)	248/639 (38.8)	71/248 (28.6)	177/248 (71.4)	
>50 y, N (%)	202/639 (31.6)	76/202 (37.6)	126/202 (62.4)	
HIV status ^c^				
Negative, N (%)	411/617 (66.6)	174/411 (42.3)	237/411 (57.7)	<0.001
Positive, N (%)	206/617 (33.4)	37/206 (18.0)	169/206 (82.0)	

^a^ The positive group includes patients with at least one positive HPV DNA detection in anal specimens. ^b^ Age data were available for 639 patients (HPV-negative N = 207, HPV positive N = 432). ^c^ HIV status data available for 617 patients.

**Table 2:** Anal HPV prevalence among HIV-negative women, HIV-negative men, and HIV-positive men.

Items	Groups			
**Patients (N = 605)**	**HIV-negative women (N = 179)**	**HIV-negative men (N = 232)**	**HIV-positive men** **(N = 194)**	***p* value**
Age (years), median (range)	43.5 (18.1–73.3)	42.6 (19.4–83.4)	45.4 (18.5–86.1)	ns
Ever HPV-positive ^a^/ total patients, N (%)	78/179 (43.6)	159/232 (68.3)	162/194 (83.5)	<0.001
Ever positive to carcinogenic HPV ^b^/ total patients, N (%)	29/179 (16.2)	54/232 (23.3)	60/194 (30.9)	0.0036
Repeatedly tested patients				
Patients with more than one HPV test, N (%)	54/179 (30.2)	83/232 (35.8)	91/194 (46.9)	0.0067
Number of repeated tests: mean (range)	3.21 (2–6)	2.74 (2–6)	3.23 (2–6)	ns
Months between repeat tests: mean (range)	14.1 (8.1–26.2)	14.2 (9.8–28.8)	16.4 (7.6–36.5)	0.085
HPV cleared ^c^, N (%)	12/27 (44.4)	19/59 (32.2)	14/71 (19.7)	0.026
Incident infection/ Reinfection ^c^, N (%)	12/27 (44.4)	34/59 (57.6)	38/71 (53.5)	
Persistence ^c^, N (%)	3/27 (11.1)	6/59 (10.2)	19/71 (26.7)	
HPV clearance vs. persistence HIV-negative women vs. HIV-negative men, OR (95% CI)	Reference	0.95 (0.22–4.67)	NA	ns
HPV clearance vs. persistence HIV-negative men vs. HIV-positive men, OR (95% CI)	NA	Reference	5.11 (1.62–16.14)	0.008
HPV genotypes In persistent infections	HPV6, **HPV16**, HPV61	HPV6, HPV11, **HPV16**(4)	HPV6(6), HPV11, **HPV16**(4), **HPV18**, **HPV58**(2), HPV61, HPV66, HPV70, HPV81, HPV85	
**Samples (N = 1100)**	**Samples from** **HIV-negative women (N = 301)**	**Samples from** **HIV-negative men** **(N = 376)**	**Samples from** **HIV-positive men** **(N = 423)**	***p* value**
HPV-positive/total samples, N (%)	110/301 (36.5%)	222/376 (59%)	320/423 (75.6%)	<0.001
Genotype detected (N)	HPV6 (20), HPV11 (3), **HPV16** (18), HPV17 ^d^, **HPV18** (3), **HPV31** (3), **HPV33**, HPV38 ^d^, HPV39, HPV53 (9), HPV54 (3), **HPV58** (8), HPV61 (6), HPV62, HPV66 (5), HPV68, HPV70 (6), HPV71, HPV81, HPV82, HPV83 (2), HPV84 (5), HPV110 (2) ^d^, non-typable genotypes (8)	HPV6 (53), HPV11 (37), **HPV16** (36), **HPV18** (6), **HPV31** (8), HPV32, **HPV33** (4), HPV35, HPV45 (7), HPV42, HPV53 (9), HPV54 (2), **HPV58** (8), HPV59, HPV61, HPV62 (5), HPV66 (3), HPV68, HPV70, HPV72 (5), HPV73 (2), HPV81 (2), HPV82, HPV83 (4), HPV84, HPV85 (4), HPV107 (2) ^d^, non-typable genotypes (13)	HPV6 (59), HPV11 (19), **HPV16** (39), **HPV18** (9), **HPV31** (9), HPV32 (6), **HPV33** (14), **HPV35**, **HPV39**, HPV44 (3), **HPV45** (7), **HPV51**, **HPV52** (2), HPV53 (12), HPV54 (7), HPV55, **HPV56**, **HPV58** (22), HPV61 (17), HPV62 (5), HPV66 (9), HPV68, HPV70 (10), HPV71, HPV72 (5), HPV73, HPV81 (4), HPV82, HPV83 (6), HPV84 (4), HPV85 (5), HPV86, HPV87, HPV97 (3), HPV102 (2), HPV107 ^d^, HPV113 ^d^, HPV120 ^d^, HPV145 ^d^, non-typable genotypes (29)	
IARC group 1: carcinogenic HPVs ^e^, N (%)	34/102 (33.3)	71/209 (34.0)	106/291 (36.4)	ns
IARC group 2A/2B: probably/possibly carcinogenic HPVs ^e^, N (%)	16/102 (15.7)	26/209 (12.4)	41/291 (14.1)	
IARC group 3 and LR HPVs ^2^, N (%)	52/102 (51.0)	112/209 (53.6)	144/291 (49.5)	
Samples positive to vaccine-preventable HPV genotypes				
Bivalent, N (%)	21/98 (21.4)	42/209 (20.1)	48/291 (16.5)	ns
Quadrivalent, N (%)	44/98 (44.9)	133/209 (63.6)	127/291 (43.6)	<0.001
Nonavalent, N (%)	56/98 (57.1)	160/209 (76.6)	181/291 (62.2)	<0.001

^a^ Patients who tested positive for HPV at least once during the study period. ^b^ Patients who tested positive for at least one IARC Group 1 carcinogenic HPV genotype (HPV16, 18, 31, 33, 35, 39, 45, 51, 52, 56, 58, and 59). These genotypes are shown in bold in the rows below. ^c^ Patients who tested positive for HPV at least once in repeated tests (number reported as the denominator for each group) were classified according to the HPV outcome as cleared if they turned to be negative, as reinfected if they tested positive for at least two different HPV genotypes in sequential samples, or as persistent if they tested positive for more than one year to the same HPV genotype. ^d^ Genotypes 17, 38, 107, 113, 120, and 145 are cutaneous HPVs of the genus Papillomaviridae beta. ^e^ IARC Group 1 “Carcinogenic to Humans” includes HPV16, 18, 31, 33, 35, 39, 45, 51, 52, 56, 58, and 59; Group 2A “Probably Carcinogenic to Humans” includes HPV68 and Group 2B HPV26, 53, 66, 67, 70, 73, 82, 30, 34, 69, 85, and 97; ^2^ all other HPV genotypes detected in this study, including beta HPVs, were included in the IARC group 3 and LR HPVs category.

## Data Availability

The datasets containing all data analyzed, supporting the results of this study, will be made available by the authors, without undue reservation.
